# Comparison of clinical characteristics among younger and elderly deceased patients with COVID-19: a retrospective study

**DOI:** 10.18632/aging.202139

**Published:** 2020-12-11

**Authors:** Xueyun Tan, Shuai Zhang, Juanjuan Xu, Mei Zhou, Qi Huang, Limin Duan, Zhilei Lv, Hui Xia, Wenjing Xiao, Zhengrong Yin, Yang Jin

**Affiliations:** 1 Department of Respiratory and Critical Care Medicine, Union Hospital, Tongji Medical College, Huazhong University of Science and Technology, Wuhan, China

**Keywords:** COVID-19, SARS-CoV-2, age, comorbidity, complication

## Abstract

We aimed to compare the age-related clinical characteristics between younger and elderly deceased COVID-19 patients. This single-center retrospective study included 163 adult deceased COVID-19 patients who were admitted to Wuhan Union Hospital West Campus from January 12, 2020, to March 30, 2020. Demographic and clinical features were collected by reviewing the medical records. The median age of the 163 deceased patients was 69 (interquartile range [IQR], 62-78) years. They were classified as younger (age 18-69 years; 86/163, 52.8%) and elderly (≥70 years; 77/163, 47.2%) subjects. Younger deceased patients were more likely to develop fever (72/86 vs 54/77, P=0.039) than elderly deceased patients were while anorexia was (29/77 vs 19/86, P=0.029) more common in elderly deceased patients than in younger deceased patients. In multivariate analyses, age was a protective factor for acute cardiac injury of deceased COVID-19 patients (odds ratio [OR] 0.968, [95% confidence interval (CI), 0.940-0.997]; P=0.033) while chronic cardiac disease was a risk factor for acute cardiac injury of deceased COVID-19 patients (OR 2.660 [95%CI, 1.034-6.843]; P=0.042). Our study described the clinical characteristics of younger and elderly deceased COVID-19 patients and demonstrated that younger deceased patients were more likely to develop an acute cardiac injury.

## INTRODUCTION

Since December 2019, an ongoing outbreak of Coronavirus disease 2019 (COVID-19) has struck Wuhan, China [1]. As of August 23, 2020, COVID-19 has affected over 23 057 288 individuals in more than 200 countries, and resulted in more than 800 000 deaths worldwide [2]. The mortality rate in China was reported to be 5.45% as of August 24, 2020 [3]. However, the mortality rate of critically ill COVID-19 patients was extremely high [4]. Many studies revealed that age and comorbidities such as hypertension and chronic cardiac disease were risk factors for mortality of COVID-19 patients [5–9]. Meanwhile, respiratory and cardiac complications were more frequent in non-survivors than in survivors [10]. A previous study indicated that COVID-19 patients order than 70 years old were more likely to hospitalized [11]. Another study, however, showed that age ≥ 70 was an independent risk factor for in-hospital death of COVID-19 patients with diabetes [12]. However, the differences between younger and elderly COVID-19 deceased patients are still unknown. This study aimed at comparing the clinical differences between younger and elderly COVID-19 deceased patients and to provide insights on the relationship between age and death in patients with COVID-19.

## RESULTS

### Clinical characteristics of younger and elderly deceased patients

As shown in Table 1, 163 deceased patients were recruited into the study. Among whom 109 were male and 54 were female. The median age of all the 163 deceased patients was 69.0 (IQR 62.0-78.0) years. Further, the patients were divided into two groups according to the median age. Of these, 86 (52.8%) patients were classified as younger (18-69 years old) subjects, and 77 (47.2%) patients were classified as elderly (≥ 70 years old) subjects. The most common presenting symptoms were fever (126 [77.3%]), dyspnea (113 [69.3%]), and cough (104 [63.8%]). Other common symptoms include fatigue, shortness of breath, sputum production, anorexia, myalgia, and diarrhea. Younger deceased patients were more likely to develop fever than elderly deceased patients (72/86 vs 54/77, P=0.039) while anorexia was more common in elderly deceased patients than in the younger deceased patients (29/77 vs 19/86, P=0.029). It was established that the median time from onset of illness to death was 25.0 (IQR 18.0-35.0) days and 22.0 (IQR 15.0-33.5) days in younger deceased patients and elderly deceased patients respectively.

**Table 1:** Clinical characteristics of younger and elderly deceased patients with COVID-19.

**Characteristics**	**Total (n=163)**	**Younger (n=86)**	**Elderly (n=77)**	**P value**
Age, years	69.0 (62.0-78.0)	62.0 (57.0-66.0)	78.0 (73.0-83.0)	<0.001*
Male Sex	109 (66.9%)	58 (67.4%)	51 (66.2%)	0.870‡
Comorbidities				
Hypertension	63 (38.7%)	27 (31.4%)	36 (46.8%)	0.044‡
Diabetes	30 (18.4%)	16 (18.6%)	14 (18.2%)	0.945‡
COPD	4 (2.5%)	0 (0.0%)	4 (5.2%)	0.048§
Chronic cardiac disease	25 (15.3%)	8 (9.3%)	17 (22.1%)	0.024‡
Chronic hepatic disease	3 (1.8%)	2 (2.3%)	1 (1.3%)	1.000§
Chronic renal disease	10 (6.1%)	3 (3.5%)	7 (9.1%)	0.193§
Cerebrovascular disease	11 (6.7%)	4 (4.7%)	7 (9.1%)	0.259‡
Malignancy	18 (11.0%)	10 (11.6%)	8 (10.4%)	0.801‡
Anemia	5 (3.1%)	3 (3.5%)	2 (2.6%)	1.000§
Symptoms on admission				
Fever	126 (77.3%)	72 (83.7%)	54 (70.1%)	0.039‡
Fatigue	92 (56.4%)	51 (59.3%)	41 (53.2%)	0.436‡
Myalgia	36 (22.1%)	20 (23.3%)	16 (20.8%)	0.704‡
Cough	104 (63.8%)	58 (67.4%)	46 (59.7%)	0.307‡
Sputum production	65 (39.9%)	33 (38.4%)	32 (41.6%)	0.678‡
Dyspnea	113 (69.3%)	57 (66.3%)	56 (72.7%)	0.373‡
Nausea	6 (3.7%)	4 (4.7%)	2 (2.6%)	0.685§
Vomiting	6 (3.7%)	4 (4.7%)	2 (2.6%)	0.685§
Abdominal pain	5 (3.1%)	3 (3.5%)	2 (2.6%)	1.000§
Diarrhea	27 (16.6%)	16 (18.6%)	11 (14.3%)	0.459‡
Anorexia	48 (29.4%)	19 (22.1%)	29 (37.7%)	0.029‡
Headache	5 (3.1%)	4 (4.7%)	1 (1.3%)	0.371§
Shortness of breath	66 (40.5%)	34 (39.5%)	32 (41.6%)	0.793‡
Days from illness onset to admission	10.0 (7.0-15.0)	11.0 (8.8-15.0)	10.0 (6.0-15.0)	0.084*
Days from illness onset to death	23.0 (17.0-34.0)	25.0 (18.0-35.0)	22.0 (15.0-33.5)	0.355*
Vital signs on admission				
Body temperature, °C	36.9 (36.5-37.8)	37.0 (36.5-38.0)	36.8 (36.5-37.5)	0.342*
Heart rate, beats/minute	90.0 (79.0-101.0)	91.5 (80.8-107.5)	88.0 (75.5-98.0)	0.017*
≥100	49 (30.1%)	32 (37.2%)	17 (22.1%)	0.035‡
<100	114 (69.9%)	54 (62.8%)	60 (77.9%)
Respiratory rate, breaths/minute	23.0 (20.0-30.0)	23.5 (20.0-30.0)	22.0 (20.0-30.0)	0.662*
≥24	75/162 (46.3%)	43/86 (50.0%)	32/76 (42.1%)	0.315‡
<24	87/162 (53.7%)	43/86 (50.0%)	44/76 (57.9%)
Systolic blood pressure, mmHg	137.0 (120.0-150.0)	131.0 (117.8-150.0)	142.0 (130.0-152.0)	0.012*
Diastolic blood pressure, mmHg	80.0 (70.0-88.0)	80.0 (70.0-89.0)	80.0 (72.0-88.0)	0.833*
SpO_2_, %	90.0 (81.0-95.0)	90.0 (82.0-95.0)	91.0 (80.0-96.0)	0.936*
≥93	61 (37.4%)	32 (37.2%)	29 (37.7%)	0.952‡
<93	102 (62.6%)	54 (62.8%)	48 (62.3%)
CURB-65 score on admission	1.0 (1.0-2.0)	1.0 (1.0-2.0)	1.0 (0-2.0)	0.436*
qSOFA score on admission	1.0 (0-1.0)	1.0 (0-1.0)	1.0 (0-1.0)	0.139*

Note: data are presented as medians (interquartile ranges, IQR), n (%) and n/N (%). P values are calculated by Wilcoxon rank-sum test, χ2 test, or Fisher’s exact test, as appropriate.

Abbreviations: COPD, chronic obstructive pulmonary disease; SpO_2_, Oxygen saturation; qSOFA, quick Sequential Organ Failure Assessment.

*Calculated using the Wilcoxon rank-sum test.

‡Calculated using the χ2 test.

§Calculated using the Fisher’s exact test.

The vital signs of the 163 deceased patients on admission are shown in Table 1. The systolic blood pressure of elderly deceased patients was higher than younger deceased patients (P=0.012). However, the heart rate of younger deceased patients was faster than that of the older deceased patients (P=0.017). No significant differences were seen in any other vital signs between the two groups. The most common comorbidities in younger deceased patients were hypertension (31.4%), diabetes (18.6%), and malignancy (11.6%), while in elderly deceased patients, hypertension (46.8%), chronic cardiac disease (22.1%) and diabetes (18.2%) were most common comorbidities. Elderly deceased patients showed more presence of hypertension (36/77 vs 27/86, P=0.044), chronic obstructive pulmonary disease (COPD) (4/77 vs 0/86, P=0.048), and chronic cardiac disease (17/77 vs 8/86, P=0.024) than younger deceased patients did. The quick Sequential Organ Failure Assessment (qSOFA) score and CURB-65 pneumonia severity score were used to evaluate the severity of pneumonia in patients on admission. No significant differences were seen between younger and elderly deceased patients using the qSOFA score and CURB-65 score.

### Laboratory and radiographic findings of younger and elderly deceased patients on admission

The laboratory findings of the 163 deceased patients on admission are shown in Table 2. 138 (84.7%) patients presented with a lymphocyte count below the normal range (1.1-3.2 × 10^9^ cells/L) and 97 (59.5%) patients had neutrophil count above the normal range (1.8-6.3 × 10^9^ cells/L). Moreover, 153 (93.7%) patients had elevated C-reactive protein (CRP) (>8mg/L) and 145 (89.0%) patients had increased lactate dehydrogenase levels (>245U/L).

**Table 2:** Laboratory findings of younger and elderly deceased patients with COVID-19 on admission.

**Characteristics**	**Total (n=163)**	**Younger(n=86)**	**Elderly(n=77)**	**P value**
White blood cell count, ×10^9^/L	7.9 (5.5-10.8)	7.4 (5.3-10.4)	8.3 (5.9-10.9)	0.302*
Red blood cell count, ×10^12^/L	4.1 (3.8-4.5)	4.1 (3.8-4.5)	4.1 (3.7-4.5)	0.400*
Hemoglobin, g/L	126.8 (± 20.0)	126.6 (± 19.1)	127.1 (± 21.2)	0.867†
Platelet count, ×10^9^/L	163.50 (116.25-231.50)	164.50 (109.50-231.50)	161.50 (122.00-232.00)	0.905*
Neutrophil count, ×10^9^/L	7.01 (4.50-9.72)	6.58 (4.31-9.30)	7.23 (4.68-9.98)	0.335*
Lymphocyte count, ×10^9^/L	0.62 (0.46-0.82)	0.57 (0.44-0.83)	0.64 (0.48-0.82)	0.309*
NLR	10.58 (6.77-16.58)	10.60 (6.56-16.76)	10.47 (6.96-16.03)	0.954*
Monocyte count, ×10^9^/L	0.30 (0.20-0.48)	0.30 (0.19-0.47)	0.30 (0.21-0.50)	0.554*
Eosinophil count, ×10^9^/L	0.00 (0.00-0.02)	0.00 (0.00-0.01)	0.01 (0.00-0.04)	0.019*
Basophil count, ×10^9^/L	0.02 (0.01-0.03)	0.02 (0.00-0.03)	0.02 (0.01-0.03)	0.916*
Total bilirubin, μmol/L	13.45 (9.50-20.73)	12.85 (9.20-20.05)	14.35 (9.83-21.30)	0.562*
Direct bilirubin, μmol/L	4.90 (3.30-8.50)	4.80 (3.30-8.35)	5.10 (3.50-8.70)	0.719*
Alanine aminotransferase, U/L	36.00 (23.25-53.75)	36.50 (25.50-55.50)	35.50 (18.50-50.00)	0.193*
Aspartate aminotransferase, U/L	44.00 (31.00-61.75)	44.00 (31.00-61.00)	43.50 (29.00-64.50)	0.621*
Alkaline phosphatase, U/L	69.50 (48.25-93.00)	72.00 (48.25-91.75)	66.00 (48.25-94.75)	0.631*
γ-glutamyl transpeptidase, U/L	41.00 (24.25-81.75)	37.00 (25.25-94.50)	44.50 (23.25-71.00)	0.973*
Total protein, g/L	61.30 (57.83-64.58)	60.95 (57.28-65.18)	61.90 (58.30-64.40)	0.798*
Albumin, g/L	28.13 (± 5.00)	27.99 (± 4.67)	28.30 (± 5.38)	0.701†
Globin, g/L	33.20 (29.15-37.53)	32.40 (29.30-37.68)	33.85 (28.95-37.50)	0.965*
Albumin/globin	0.80 (0.70-1.00)	0.85 (0.70-1.00)	0.80 (0.70-1.00)	0.890*
Prealbumin, mg/L	85.45 (65.83-126.88)	88.20 (69.10-129.10)	83.80 (58.75-121.85)	0.117*
Total bile acid, μmol/L	3.20 (2.00-5.70)	3.05 (1.75-4.40)	3.75 (2.18-6.60)	0.056*
Creatinine, μmol/L	75.4 (63.35-95.70)	71.90 (56.93-87.98)	80.95 (71.10-112.15)	0.001*
Blood urea nitrogen, mmol/L	7.02 (5.06-10.47)	6.91 (4.41-9.16)	7.94 (5.43-13.94)	0.016*
Uric acid, μmol/L	235.05 (173.25-331.03)	211.10 (158.75-272.70)	273.95 (199.38-381.05)	<0.001*
Glucose, mmol/L	7.57 (6.26-11.02)	7.93 (6.50-11.78)	6.99 (5.87-10.07)	0.066*
Creatine kinase, U/L	132.00 (68.50-262.00) (n=134)	111.00 (71.00-207.25) (n=72)	160.00 (57.50-311.75) (n=62)	0.326*
Lactate dehydrogenase, U/L	476.00 (358.00-591.00)	497.00 (381.00-597.75)	472.00 (338.00-586.00)	0.604*
C-reactive protein, mg/L	68.88 (± 43.86)	69.73 (± 44.45)	67.92 (± 43.47)	0.799†
Procalcitonin, ng/mL	0.26 (0.14-0.45) (n=114)	0.23 (0.11-0.41) (n=60)	0.35 (0.14-0.76) (n=54)	0.075*
D-dimer, μg/mL	2.31 (0.68-8.00) (n=137)	2.68 (0.81-8.00) (n=72)	2.07 (0.58-8.00) (n=65)	0.441*
Prothrombin time, seconds	14.20 (13.00-15.30) (n=147)	14.20 (12.90-15.40) (n=79)	14.20 (13.10-15.10) (n=68)	0.529*
International normalized ratio	1.12 (1.00-1.24) (n=148)	1.12 (1.00-1.24) (n=79)	1.12 (1.01-1.24) (n=69)	0.470*
Activated partial thromboplastin time, seconds	39.19 (± 8.78) (n=149)	37.65 (± 7.89) (n=79)	40.93 (± 9.45) (n=70)	0.022†
Fibrinogen, g/l	4.49 (3.20-5.25) (n=149)	4.53 (3.20-5.38) (n=79)	4.44 (3.20-5.25) (n=70)	0.666*
Thrombin time, seconds	15.80 (14.95-17.25) (n=149)	15.70 (14.90-16.90) (n=79)	15.80 (15.00-17.30) (n=70)	0.725*
Brain natriuretic peptide, pg/ml	98.60 (39.90-185.40) (n=89)	95.50 (39.60-144.80) (n=47)	102.75 (41.15-206.13) (n=42)	0.562*
Myoglobin, ng/ml	127.80 (62.85-287.05) (n=85)	98.85 (48.90-222.63) (n=44)	171.70 (85.25-548.60) (n=41)	0.015*
Creatine kinase-MB, ng/ml	1.70 (0.90-5.73) (n=92)	1.50 (0.80-4.80) (n=49)	3.00 (0.90-7.00) (n=43)	0.120*
Hypersensitive cardiac troponin Ι, ng/L	23.10 (8.78-114.18) (n=92)	15.30 (7.30-86.40) (n=47)	41.60 (12.75-436.65) (n=45)	0.017*

Note: data are presented as mean (± SD), medians (interquartile ranges, IQR) and medians (interquartile ranges, IQR) (n). P values are calculated by Wilcoxon rank-sum test or independent group t test, as appropriate.

Abbreviations: NLR, neutrophil-to-lymphocyte ratio.

*Calculated using the Wilcoxon rank-sum test.

†Calculated using the independent group t test.

The blood eosinophil count of younger deceased patients was much lower than elderly deceased patients (P=0.019). Compared to younger deceased patients, elderly deceased patients had notably higher levels of creatinine (P=0.001), blood urea nitrogen (P=0.016), uric acid (P<0.001), myoglobin (P=0.015), and hypersensitive cardiac troponin Ι (P=0.017) than younger deceased patients did. Moreover, the activated partial thromboplastin time of elderly deceased patients was significantly longer than that of younger deceased patients (P=0.022).

Besides, a chest CT scan was conducted on 74 younger and 65 elderly deceased patients four days before or after hospitalization (Table 3). 116 (83.5%) deceased patients showed bilateral lung involvement and 113 (81.3%) patients had a lung involvement ratio of more than 30%. The most common findings on chest CT scans were ground-glass opacity (128/139, 92.1%) and consolidation (125/139, 89.9%).

**Table 3:** CT findings of younger and elderly deceased patients with COVID-19 on admission.

**Characteristics**	**Total (n=139)**	**Younger (n=74)**	**Elderly(n=65)**	**P value**
Lung involvement ratio (≥30%)	113 (81.3%)	58 (78.4%)	55 (84.6%)	0.347‡
Bilateral lung involvement	116 (83.5%)	60 (81.1%)	56 (86.2%)	0.422‡
Ground-glass opacity	128 (92.1%)	69 (93.2%)	59 (90.8%)	0.590‡
Consolidation	125 (89.9%)	65 (87.8%)	60 (92.3%)	0.382‡
Pleural effusion	29 (20.9%)	12 (16.2%)	17 (26.2%)	0.150‡
Lymphadenopathy	2 (1.4%)	1 (1.4%)	1 (1.5%)	>0.999§

Note: data are presented as n (%). P values are calculated by χ2 test or Fisher’s exact test, as appropriate.

‡Calculated using the χ2 test.

§Calculated using the Fisher’s exact test.

### Univariate and multivariate analysis for complications in younger and elderly deceased patients

The most commonly observed complication, among the 163 deceased patients, was acute respiratory distress syndrome (ARDS) (97.5%), followed by respiratory failure (74.2%), acute cardiac injury (36.8%), septic shock (34.4%), and acute kidney injury (21.5%) (Table 4). The comparison between complications in younger and elderly deceased patients is shown in Table 4. There were no significant differences observed in complications between younger and elderly deceased patients.

**Table 4:** Complications of younger and elderly deceased patients with COVID-19.

**Complications**	**Total (n=163)**	**Younger (n=86)**	**Elderly (n=77)**	**P value**
ARDS	159 (97.5%)	84 (97.7%)	75 (97.4%)	>0.999§
Respiratory failure	121 (74.2%)	66 (76.7%)	55 (71.4%)	0.439‡
Septic shock	56 (34.4%)	25 (29.1%)	31 (40.3%)	0.133‡
Acute cardiac injury	60 (36.8%)	37 (43.0%)	23 (29.9%)	0.082‡
Acute kidney injury	35 (21.5%)	18 (20.9%)	17 (22.1%)	0.859‡
Liver injury	25 (15.3%)	12 (4.0%)	13 (16.9%)	0.604‡
Bacterial infections	24 (14.7%)	13 (15.1%)	11 (14.3%)	0.881‡
Fungal infections	13 (8.0%)	4 (4.7%)	9 (11.7%)	0.098‡

Note: data are presented as n (%). P values are calculated by χ2 test or Fisher’s exact test, as appropriate.

Abbreviations: ARDS, acute respiratory distress syndrome.

‡Calculated using the χ2 test.

§Calculated using the Fisher’s exact test.

Subsequently, we analyzed the factors associated with acute cardiac injury and septic shock in COVID-19 deceased patients by using a logistic regression model. In multivariable analyses, age was a protective factor for acute cardiac injury of COVID-19 deceased patients (odds ratio [OR] 0.968, [95% confidence interval (CI), 0.940-0.997]; P=0.033) while the chronic cardiac disease was a risk factor for acute cardiac injury of COVID-19 deceased patients (OR 2.660 [95%CI, 1.034-6.843]; P=0.042) (Table 5). Bacterial infections (aOR 66.304, [95% CI, 8.490-517.785]; P<0.001) and fungal infections (aOR 8.946, [95% CI, 1.654-48.380]; P=0.011) were also associated with an increased likelihood of septic shock. However, age was not associated with septic shock in COVID-19 deceased patients (aOR 1.013, [95% CI, 0.979-1.048]; P=0.462) (Table 6).

**Table 5:** Univariable and multivariate analyses in factors associated with acute cardiac injury.

**Factors**	**Univariable OR (95% CI)**	**P value**	**Multivariate OR (95% CI)**	**P value**
Age#	0.979 (0.953-1.005)	0.116	0.968 (0.940-0.997)	0.033
Sex (male vs female)	1.608 (0.800-3.232)	0.183	1.451 (0.696-3.025)	0.321
Hypertension (yes vs no)	1.523 (0.795-2.917)	0.205	1.651 (0.798-3.415)	0.177
Chronic cardiac injury (yes vs no)	2.098 (0.888-4.957)	0.091	2.660 (1.034-6.843)	0.042
Diabetes (yes vs no)	0.689 (0.293-1.621)	0.394	0.695 (0.278-1.735)	0.436

Note: # Per 1 unit increase.

Abbreviations: OR, odds ratio; CI, confidence interval.

**Table 6:** Univariable and multivariate analyses in factors associated with septic shock.

**Factors**	**Univariable OR (95% CI)**	**P value**	**Multivariate OR (95% CI)**	**P value**
Age#	1.017 (0.990-1.046)	0.222	1.013 (0.979-1.048)	0.462
Bacterial infections (yes vs no)	73.879 (9.607-568.107)	<0.001	66.304 (8.490-517.785)	<0.001
Fungal infections (yes vs no)	12.833 (2.733-60.257)	0.001	8.946 (1.654-48.380)	0.011

Note: # Per 1 unit increase.

Abbreviations: OR, odds ratio; CI, confidence interval.

## DISCUSSION

Several studies reported that age was an independent risk factor of mortality in patients with COVID-19 [6–9, 13]. This study found that younger deceased patients were more likely to develop fever than elderly deceased patients were. Anorexia was, however, more common in elderly deceased patients than younger deceased patients. We also found that younger deceased patients were more likely to develop an acute cardiac injury.

The mortality rate in our study was at 12.12%, which is much higher than the current national reports (4634/84981, 5.45%) [3]. This might be related to the following reasons. First, Union Hospital West Campus was a designed hospital for severely or critically ill patients with COVID-19 in Wuhan, China. Some severely or critically ill patients were transferred from other hospitals, which led to a relatively high mortality rate. Secondly, as the spread of the virus was well controlled in the late stage and with improved virus detection rate, patients with COVID-19 could be treated in a timely and effective manner.

A previous study demonstrated that the infection fatality ratio for individuals 70 years and older was 10.5% while for those patients younger than 70 years was 0.43% [14]. Age-related diseases and vulnerability to COVID-19 are highly intertwined [15]. Indeed, this study showed that elderly deceased patients presented with hypertension, COPD, and chronic cardiac disease more frequently. Previous studies showed that the most commonly observed comorbidity in patients with COVID-19 was hypertension [16, 17]. This is similar to our study. However, considering that age was reported to be an independent risk factor of poor outcomes in patients with COVID-19 [6–9, 13], aging, and its related mechanisms may play an important role in disease severity of COVID-19.

The most common symptoms of the 163 deceased patients were fever, dyspnea, cough, fatigue, and shortness of breath, which were similar to other researches [18, 19]. However, in our study, fever was more frequent in younger deceased patients while anorexia was more frequent in elderly deceased patients. These results indicate that there might be some differences in disease pathogenesis and progression between the younger and elderly deceased patients. A possible hypothesis is that in critically ill COVID-19 patients, cellular hyperfunctions of elderly deceased patients were more likely to switch to cellular exhaustion, and loss of functions at late stages [15].

The main cause of death in both groups was ARDS and respiratory failure. However, acute cardiac injury also plays an important role in in-hospital mortality in patients with COVID-19 [20]. The mechanisms of cardiac injury are however not yet well established, but are likely to involve increased cardiac stress due to respiratory failure and hypoxemia, direct myocardial infection by SARS-CoV-2, indirect injury from the systemic inflammatory response, or a combination of all three factors [21, 22]. It has been reported that SARS-CoV-2 could be detected within the myocardium of deceased patients but the presence of SARS-CoV-2 was not associated with an influx of inflammatory cells [23]. These results might suggest that myocardial injury is likely related to systemic consequences rather than direct damage caused by SARS-CoV-2 [24]. It has been reported that older COVID-19 patients were more likely to develop an acute cardiac injury [25]. However, a previous study demonstrated that cardiovascular comorbidities increased relative risks for adverse outcomes most substantially in the younger COVID-19 patients with progressively diminishing relative effects in older ages [26]. In our study, we found that age was a protective factor for the acute cardiac injury of deceased patients. These results indicated that despite the lower incidences of acute cardiac injury in younger patients, it is vital to prevent acute cardiac injury in younger patients. Moreover, once a younger patient developed an acute cardiac injury, more attention should be paid to prevent the adverse outcomes caused by the cardiac injury.

Our study had several limitations. Firstly, it was retrospective and all data was collected from case records. In addition, some important indicators such as the immunological index, and treatment history of patients were not collected in our study. Secondly, ours is a single-center study, although the health-care workers were from all over the country, hence some factors including the bacteria colonizing the hospital might be different. Thirdly, Union Hospital West Campus was a designated hospital for severely or critically ill COVID-19 patients in Wuhan, China. Consequently, there might be some selection bias, and therefore the external validity of the conclusions needs to be further validated.

In summary, this study described the clinical characteristics of younger and elderly deceased COVID-19 patients. We established that younger deceased patients were more likely to develop fever than elderly deceased patients were while anorexia was more common in elderly deceased patients than in younger deceased patients. The multivariate logistic regression analysis showed that younger deceased patients were more likely to develop an acute cardiac injury. Moreover, this study demonstrated that it is important to monitor cardiac function and prevent acute cardiac injury in severely and critically ill patients with COVID-19 regardless of age.

## MATERIALS AND METHODS

### Study design and participants

This single-center, retrospective study was conducted at Wuhan Union Hospital West Campus, a designated hospital for severely and critically ill COVID-19 patient treatment. This study was approved by the Institutional Ethics Committee of Union Hospital, Tongji Medical College, Huazhong University of Science and Technology (NO.20200036), and the requirement for informed consent was exempted by the Ethics Committee.

**Figure 1:**
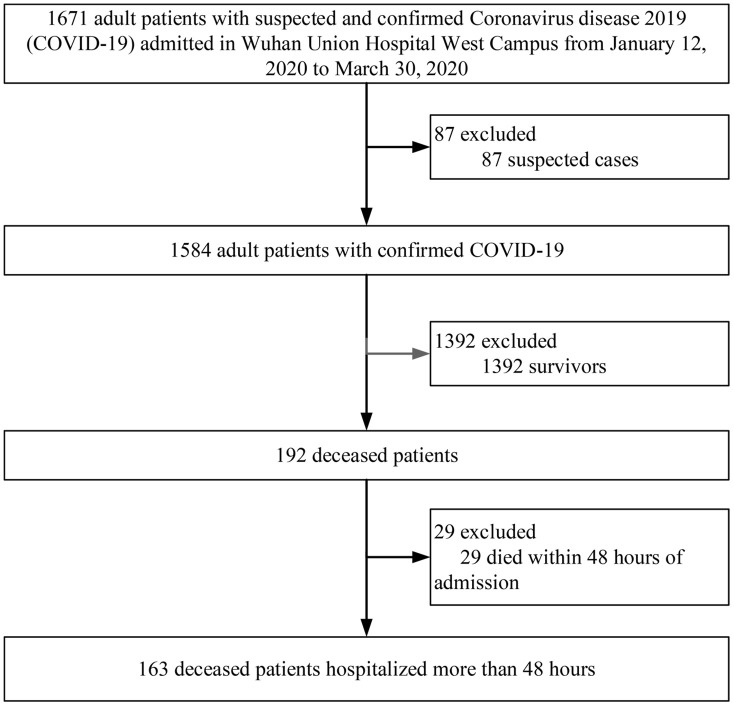
**Flow diagram of patient selection.**

The inclusion criteria were adult deceased patients with confirmed COVID-19 who had been hospitalized for more than 48 hours. From January 12, 2020, to March 30, 2020, a total of 1671 adult patients with suspected and confirmed COVID-19 were admitted to Wuhan Union Hospital West Campus. Among them, 1584 were confirmed cases, 192 (12.12%, [95% confidence interval (CI), 10.60%-13.82%]) patients with confirmed COVID-19 died until April 22, 2020. Among the 192 deceased patients, 29 died within 48 hours of admission and were excluded from the study. Finally, a total of 163 deceased patients were enrolled in this study (Figure 1).

### Definitions

The diagnosis of COVID-19 was based on the guidelines of the diagnosis and treatment of new coronavirus pneumonia (version 7) published by the National Health Commission of China [27]. Acute respiratory distress syndrome (ARDS) was diagnosed according to the Berlin Definition [28]. Septic shock was defined according to The Third International Consensus Definitions for Sepsis and Septic Shock (Sepsis-3) [29]. Acute kidney injury was diagnosed according to the KDIGO clinical practice guidelines [30]. Acute cardiac injury was diagnosed according to the COVID-19 rapid guideline: acute myocardial injury [31]. Patients with Brain Natriuretic Peptide (BNP) level above 250 pg/ml were also diagnosed as having a cardiac injury [32]. Acute liver injury was diagnosed as previously described [10].

### Data collection

We collected all available information from the medical records in the hospital. This included the epidemiological history, clinical, laboratory, radiological characteristics, and outcomes. All data collected from electronic medical records were then recorded into a standardized form and checked by professional clinicians to verify the accuracy of the data.

### Factors enrolled in multivariate analyses

Least Absolute Shrinkage and Selection Operator (LASSO) regression was applied to minimize the potential collinearity of variables measured from the same patient and over-fitting of variables as previously described [33]. In LASSO regression, the performance of the model was augmented with 10-fold cross-validation and the covariates were selected by the minimum (λ min). This regression model penalizes the absolute size of the coefficients of a regression model based on the value of λ. With larger penalties, the estimates of weaker factors shrink toward zero, so that only the strongest factors remain in the model. For respiratory failure, six variables (age, sex, COPD, hypertension, chronic cardiac disease, and diabetes) were selected to enroll into the LASSO regression model but no variables were available for further analysis. For acute cardiac injury, five variables (age, sex, hypertension, chronic cardiac disease, and diabetes) were selected to enroll into the LASSO regression model. All five variables were selected for further multivariable analysis. For septic shock, five variables (age, sex, bacterial infections, fungal infections, and diabetes) were selected to enroll into the LASSO regression model. Age, bacterial infections, and fungal infections were selected for further multivariable analysis. For acute kidney injury, six variables (age, sex, chronic kidney disease, hypertension, chronic cardiac disease, and diabetes) were selected to enroll into the LASSO regression model but no variables were available for further analysis. Other complications including liver injury and gastrointestinal bleeding were not available for multivariable analysis.

### Statistical analysis

Categorical variables were presented as frequency rates and percentages, and continuous variables were expressed as mean ± standard deviation (SD) if they were normally distributed or median (interquartile range [IQR]) if they were not. Proportions for categorical variables were compared using the χ2 test or Fisher’s exact test. Besides, means for continuous variables were compared using independent group t test when the data was normally distributed. Otherwise, the Wilcoxon rank-sum test was employed. A 95% confidence interval (CI) of mortality was analyzed by Wilson Score CI. All statistics were two-tailed and a P value less than 0.05 was considered as significant. All statistical analyses were performed using SPSS software (version 22.0).
